# Analysis of major and minor IAS-USA PI mutations in the MONET trial of darunavir/ritonavir monotherapy versus DRV/r + 2NRTIs

**DOI:** 10.1186/1758-2652-13-S4-P131

**Published:** 2010-11-08

**Authors:** F Pulido, JR Arribas, A Hill, Y van Delft, C Moecklinghoff

**Affiliations:** 1 Hospital 12 de Octubre, Madrid, Spain; 2 Hospital la Paz, Madrid, Spain; 3 Liverpool University and Tibotec BVBA, Liverpool, UK; 4 Janssen-Cilag, Tilburg, Netherlands; 5 Janssen-Cilag, Neuss, Germany

## Background

For patients on treatment with HIV RNA <50 copies/mL, it is unknown whether the genetic barrier to evolution of resistance is different for DRV/r monotherapy, compared with standard triple combinations of antiretrovirals. Several minor IAS-USA mutations are detected frequently in samples from PI naïve patients.

## Methods

In the MONET trial, 256 patients with no history of virological failure and HIV RNA <50 copies/mL on current HAART (NNRTI based (43%), or PI based (57%) switched to either DRV/r monotherapy (800/100 mg OD) versus DRV/r + 2NRTIs. HIV RNA levels were evaluated at Weeks 2, 12, 24, 36, 48, 60, 72, 84 and 96: all patient samples with HIV RNA above 50 copies/mL were sent for genotypic resistance analysis (VircoTYPE HIV-1, Beerse, Belgium). Virtual phenotype was also assessed when PI mutations were detected. The percentage of patients with major or minor IAS-USA PI mutations was analysed by treatment arm.

## Results

Patients were 81% male and 91% Caucasian, with median age 43 years, and median CD4 count of 575 cells/uL. While patients were receiving randomised treatment, HIV RNA was above 50 copies/mL for 47/1051 (4.5%) patient-visits in the DRV/r + 2NRTI arm and 69/1009 (6.8%) patient-visits in the DRV/r monotherapy arm. Of 48 patients with at least one successful genotype (27 in the DRV/r monotherapy arm, 21 in the DRV/r + 2NRTI arm), two showed major IAS-USA PI mutations during short-term elevations in HIV RNA (one per treatment arm). Both patients remained phenotypically sensitive to darunavir, with sustained HIV RNA<50 copies/mL during the trial and no change in antiretroviral treatment. The five most common minor IAS-USA mutations detected in the DRV/r mono and DRV/r + 2NRTI arms were L63P (78%, 62% respectively), I93L (59%, 19%), V77I (33%, 43%), I62V (22%, 33%) and I64V (15%, 24%). These five mutations were also commonly observed in the Stanford HIV database of 7601 samples from PI naive patients. Fourteen patients in the DRV/r monotherapy arm had repeated genotypes during intermittent low-level viraemia — there was no evidence for evolution of minor IAS-USA PI mutations over time in these patients.

## Conclusions

After 96 weeks of treatment in the MONET trial, there is no evidence for an increased risk of emergence of major or minor IAS-USA PI mutations with DRV/r monotherapy, compared to DRV/r + 2 NRTIs, and no evidence for evolution of PI mutations after repeated genotyping.

